# A critical review of manual therapy use for headache disorders: prevalence, profiles, motivations, communication and self-reported effectiveness

**DOI:** 10.1186/s12883-017-0835-0

**Published:** 2017-03-24

**Authors:** Craig S. Moore, David W. Sibbritt, Jon Adams

**Affiliations:** University of Technology Sydney, Faculty of Health, Building 10, Level 8, 235-253 Jones St, Ultimo, Sydney, NSW, 2007 Australia

**Keywords:** Headache, Migraine, Tension headache, Cervicogenic headache, Manual therapy, Physical therapy, Chiropractic, Osteopathy, Massage

## Abstract

**Background:**

Despite the expansion of conventional medical treatments for headache, many sufferers of common recurrent headache disorders seek help outside of medical settings. The aim of this paper is to evaluate research studies on the prevalence of patient use of manual therapies for the treatment of headache and the key factors associated with this patient population.

**Methods:**

This critical review of the peer-reviewed literature identified 35 papers reporting findings from new empirical research regarding the prevalence, profiles, motivations, communication and self-reported effectiveness of manual therapy use amongst those with headache disorders.

**Results:**

While available data was limited and studies had considerable methodological limitations, the use of manual therapy appears to be the most common non-medical treatment utilized for the management of common recurrent headaches. The most common reason for choosing this type of treatment was seeking pain relief. While a high percentage of these patients likely continue with concurrent medical care, around half may not be disclosing the use of this treatment to their medical doctor.

**Conclusions:**

There is a need for more rigorous public health and health services research in order to assess the role, safety, utilization and financial costs associated with manual therapy treatment for headache. Primary healthcare providers should be mindful of the use of this highly popular approach to headache management in order to help facilitate safe, effective and coordinated care.

## Background

The co-occurrence of tension headache and migraine is very high [1]. Respectively, they are the second and third most common disorders worldwide with migraine ranking as the seventh highest specific cause of disability globally [2] and the sixteenth most commonly diagnosed condition in the US [3]. These common recurrent headache disorders place a considerable burden upon the personal health, finances and work productivity of sufferers [3–5] with migraine further complicated by an association with cardiovascular and psychiatric co-morbidities [6, 7].

Preventative migraine drug treatments include analgesics, anticonvulsants, antidepressants and beta-blockers. Preventative drug treatments for tension-type headaches can include analgesics, NSAIDs, muscle relaxants and botulinum toxin as well as anticonvulsants and antidepressants. While preventative drug treatments are successful for a significant proportion of sufferers, headache disorders are still reported as under-diagnosed and under-treated within medical settings [8–16] with other studies reporting sufferers can cease continuing with preventative headache medications long-term [9, 17].

There is a number of non-drug approaches also utilized for the prevention of headaches. These include psychological therapies such as cognitive behavioral therapy, relaxation training and EMG (electromyography) biofeedback. In addition, there is acupuncture, nutritional supplementation (including magnesium, B12, B6, and Coenzyme Q10) and physical therapies. The use of physical therapies is significant, with one recent global survey reporting physical therapy as the most frequently used ‘alternative or complementary treatment’ for headache disorders across many countries [18]. One of the most common physical therapy interventions for headache management is manual therapy (MT), [19–21] which we define here as treatments including ‘spinal manipulation (as commonly performed by chiropractors, osteopaths, and physical therapists), joint and spinal mobilization, therapeutic massage, and other manipulative and body-based therapies’ [22].

Positive results have been reported in many clinical trials comparing MT to controls [23–27], other physical therapies [28–30] and aspects of medical care [31–34]. More high quality research is needed however to assess the efficacy of MT as a treatment for common recurrent headaches. Recent systematic reviews of randomized clinical trials of MT for the prevention of migraine report a number of significant methodological short-comings and the need for more high quality research before any firm conclusions can be made [35, 36]. Recent reviews of MT trials for tension-type headache and cervicogenic headache are cautious in reporting positive outcomes and the strong need for further robust research [37–41]. Despite the limited clinical evidence there has been no critical review of the significant use of MT by headache populations.

## Methods

The aim of this study is to report from the peer-reviewed literature; 1) the prevalence of MT use for the treatment of common recurrent headaches and 2) factors associated with this use across several key themes. The review further identifies key areas worthy of further research in order to better inform clinical practice, educators and healthcare policy within this area.

### Design

A comprehensive search of peer-reviewed articles published in English between 2000 and 2015 reporting new empirical research findings of key aspects of MT use among patients with migraine and non-migraine headache disorders was undertaken. Databases searched were MEDLINE, AMED, CINAHL, EMBASE and EBSCO. The key words and phrases used were: ‘headache’, ‘migraine’, ‘primary headache’, ‘cephalgia’, ‘chronic headache’ AND ‘manual therapy’, ‘spinal manipulation’, ‘manipulative therapy’, ‘spinal mobilization’, ‘chiropractic’, ‘osteopathy’, ‘massage’, ‘physical therapy’ or ‘physiotherapy’ AND then ‘prevalence’, ‘utilization’ or ‘profile’ was used for additional searches against the previous terms. The database search was accompanied by a hand search of prominent peer-reviewed journals. All authors accessed the reviewed literature (data) and provided input to analysis.

Due to the focus of the review, literature reporting randomized control trials and similar clinical research designs were excluded as were articles identified as letters, correspondence, editorials, case reports and commentaries. Further searches were undertaken of the bibliographies in the identified publications. All identified articles were screened and only those reporting new empirical findings on MT use for headache in adults were included in the review. Articles identified and selected for the review were research manuscripts mostly within epidemiological and health economics studies. The review includes papers reporting MT use pooled with the use of other therapies, but only where MT patients comprised a large proportion (as stated) of the included study population. Results were imported into Endnote X7 and duplicates removed.

### Search outcomes, analyses and quality appraisal

**Fig. 1:**
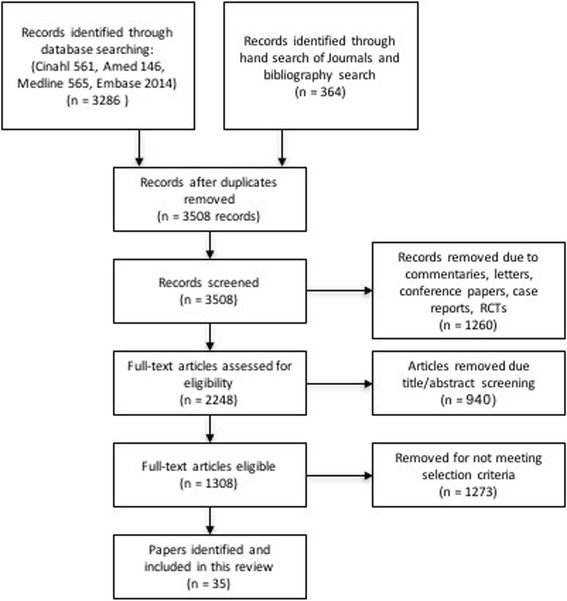
Flow chart of study selection

Figure 1 outlines the literature search process. The initial search identified 3286 articles, 35 of which met the inclusion criteria. Information from each article was organized into a review table (Table 1) to summarise the findings of the included papers. Information is reported under two selected headache groups and within each individual MT profession - chiropractic, physiotherapy, osteopathy and massage therapy – where sufficient detail was available.

**Table 1:** Research-based studies of manual therapy use for headache disorders

Authors Year	Country/ Region	Population/ Profession	Study Method	Sample size	Themes †	Prevalence use based on Headache Groupings
Ailliet et al 2010 [65]	Europe (Belgium)	Manual Therapy population/Chiropractic	Postal questionnaire by chiropractors	517 patients	1	Headache: Chiropractic 1.9%
Bethell et al 2013 [76]	North America	General population	Secondary analysis of national survey	2411	2	
Bigal et al 2008 [19]	North America	General population	Longitudinal study following a cohort of headache sufferers	Chronic migraine (520), Episodic migraine (9424)	1	Chronic migraine: Chiropractic 36.2% Physiotherapist 13.3% Episodic migraine: Chiropractic 25.7%, Physiotherapy 4.2%
Brown et al 2014 [61]	Australia	Manual Therapy population/Chiropractic	Cross-sectional survey completed by patients	486	1	Headaches: Chiropractic 5.5%
Cherkin et al 2002 [62]	North America	Manual Therapy population/Chiropractic	Practitioner completed questionnaire	2550	1	Headaches: Chiropractic Massachusetts 4.6%, Arizona 6.4%
Cooke et al 2010 [49]	North America	General population	Telephone survey to public	1210	1	Migraine: Chiropractic 6%, Massage 2%, Osteopathy 1%
Coulter et al 2002 [66]	North America	Manual Therapy population/Chiropractic	Patient questionnaires	1275	1	Headaches: Chiropractic 4.0%
Brown et al, 2013 [69]	Australia	Manual Therapy population/Chiropractic	Cross-sectional general population survey questionnaire	757	1	Headache: Chiropractic 45.5%
French et al 2013 [64]	Australia	Manual Therapy population/Chiropractic	Cross-sectional observational practitioner survey	4464	1	Headaches: Chiropractic 4%
Gaul et al 2009 [70]	Europe (Germany/Austria)	Headache clinic population	Questionnaire based patient survey	432	1,2,3,4	Mixed primary headaches: Massage 46.1%, Physiotherapy 27.8%
Gaul et al 2011 [72]	Europe (Germany/Austria)	Headache clinic population	Questionnaire-based survey	448	1,2	Migraine (78.5%): Physiotherapy 18.7%, Massage 56.4%
Gaumer G 2006 [56]	North America	General population	Random telephone survey	800	1	Headaches: Chiropractic 5.3%
Goksel et al 2014 [73]	Europe (Turkey)	Headache clinic population	Patient questionnaire through interview	110	1,2,4	Migraine (64.6%): Massage 51%
Hartvigsen et al 2003 [68]	Europe (Denmark)	Manual Therapy/Chiropractic	Questionnaire data collected by practitioners	1897 patients	1	Headache: Chiropractic 4%
Jackson P 2001 [63]	North America	Manual Therapy population/Chiropractic	Postal questionnaire to chiropractors	1500	1	Headaches: Chiropractic 15.4%
Kristoffersen et al 2012 [20]	Europe (Norway)	General population	Cross-sectional epidemiological survey	405	1,2	All Primary Headaches: Chiropractic 28% Physiotherapy 52%
Kristoffersen et al 2013 [79]	Europe (Norway)	General population	Cross-sectional epidemiological postal survey and clinical interview	253 primary 82 secondary	4	
Lambert et al 2010 [77]	Europe (UK)	Headache clinic population	Self-administered questionnaire	92	2,3	
Lyngberg et al 2005 [1, 52]	Europe (Denmark)	General population	Medical doctor interviews	740	1	Mostly migraine: Chiropractic 9% Physiotherapy 5%
Malone et al 2012 [71]	North America	General population	On-line survey via migraine website	2735	1	Migraine: Massage 29.7%
Minen et al 2014	North America	Headache clinic population	Secondary analysis of baseline questionnaire data	225	1,2	Migraine with/without aura: Chiropractic 27.1%, Massage 18.2%, Physiotherapy 4.9%
Morin et al 2014 [54]	North America (Quebec)	Manual Therapy population	Prospective survey	1402	1	Migraine: Osteopathy 1.7% Headaches: Osteopathy 2.7%
Ndetan et al 2009 [57]	North America	General population	Secondary Survey analysis	31248	1	Headache: Chiropractic 15.1%
Orrock P 2009 [75]	Australia	Manual Therapy population/Osteopathy	Mailed practitioner questionnaire	2238 patient records	1	Headache: Osteopathy 10%
Ossendorf et al 2009 [60]	Europe (Germany)	Pain clinic population	Physician-administered structured interview and questionnaires	288 (136 with Headache)	1,4	Headache: Chiropractic 22%, Physiotherapy 35%, Osteopathy 9%, Massage 54%
Rossi et al 2005 [53]	Europe (Italy)	Headache clinic population	Physician-administered structured interview	481	1,2,3,4	Migraine: Massage 10.1%, Chiropractic 8.9%, Osteopathy 2.7%
Rossi et al 2006 [58]	Italy	Headache clinic population	Physician-administered structured interview	110	1,2,3,4	Headache (CTTH): Chiropractic 21.9%, Massage 17.8%
Rossi et al 2008 [59]	Europe (Italy)	Headache clinic population	Physician administered structured interview	100	1,2,4	Headache (cluster): Chiropractic 12%, Acupressure 12%
Rubinstein et al 2000 [67]	Europe (Netherland)	Manual Therapy population/Chiropractic	Retrospective patient questionnaires	833	1	Headache: Chiropractic 7%
Sanderson et al 2013 [21]	USA, Canada, UK, Germany, France and Australia	General population	Web-based screening questionnaire	16663	1,3	Chronic migraine: 10% USA Canada 10%, France/UK 0%, Germany 1%, Australia 14% Episodic Migraine: USA 7% Canada 4%, France/UK 1%, Germany 6%, Australia 14%
S von Peter et al 2002 [78]	North America	Headache clinic population	Patient interview using a standardized questionnaire	73	2,3,4	Tension, Migraine (27%) and other headaches: Chiropractic 15.1%, Massage 42.5%
Vukovic et al 2010 [48]	Europe (Croatia)	General population	Random cross-sectional survey questionnaire	616	1	Migraine: Chiropractic 9.5%, Physiotherapy 19.4% Tension headache: Chiropractic 4.0%, Physiotherapy 12.2%
Wells et al 2010 [51]	North America	General population	National cross-sectional survey sample	23,393	1	Migraine 18.5% and Headaches 15.7%: Chiropractic/massage pooled
Wells et al 2011 [50]	North America	General population	National cross-sectional survey sample	23,393	1,3	Migraine: Chiropractic 15.4%, Massage 15.1%
Xue et al 2008 [55]	Australia	General population	Cross-sectional telephone survey	1067	1	Headaches: Chiropractic 9.3%

Themes †: 1 = MT prevalence use, 2 = Profile and motivations, 3 = Concurrent use, 4 = Self-reported effectiveness

An appraisal of the quality of the articles identified for review was conducted using a quality scoring system (Table 2) developed for the critical appraisal of health literature used for prevalence and incidence of health problems [42] adapted from similar studies [43–45]. This scoring system was applicable to the majority of study designs involving surveys and survey-based structured interviews (29 of the 35 papers) but was not applicable to a small number of included studies based upon clinical records, secondary analysis or practitioner characteristics.

**Table 2:** Description of quality criteria and scoring for selected studies

Dimensions of Quality Assessment	Points Awarded†
Methodology	
A. Sampling strategy reported/appropriate to study design	1
B. Sample size >100	1
C. Response rate >75%	1
D. Low recall bias (prospective data collection or retrospective data collection within past 12 months)	1
Reporting of Participants characteristics	
E. Classification of migraine or headache type(s) reported	1
F. Age and sex	1
G. Ethnicity	1
H. Indicator of socioeconomic status (income, education)	1
Reporting of relevant MT factors	
I. Reporting of MT use for headache	1
J. Reporting of MT financial costs	1

†Maximum score of 10 points for studies applicable to this scoring system with each item weighted equally with 0 (criterion not fulfilled) or 1 (criterion fulfilled) point

Two separate authors (CM and JA) independently searched and scored the articles. Score results were compared and any differences were further discussed and resolved by all the authors. The quality score of each relevant article is reported in Table 3.

**Table 3:** Quality score for selected studies

Dimensions of Quality Assessment				
Authors/Year	Methodology	Participant characteristics	Reporting of MT use	Total score
Ailliet et al, 2010 [65]	A, B, C	F, H	I	6
Bigal et al, 2008 [19]	A, B, C, D	E, F, G, H		8
Brown et al, 2013 [69]	A, B, C, D	F, H		6
Brown et al, 2014 [61]	A, B, C, D	F, G, H	I	8
Cherkin et al, 2002 [62]	A, B, C, D	F, G	I	7
Cooke et al, 2010 [49]	A, B, D	E, F,		5
Coulter et al, 2002 [66]	A, B, D	F, G, H		6
French et al, 2013 [64]	A, B, D	F, G, H	I	7
Gaul et al, 2009 [70]	A, B, D	E, F, G, H	I	8
Gaul et al, 2011 [72]	A, B, D	E, F, H	I	7
Gaumer G, 2006 [56]	A, B, D	F, H		5
Goksel et al, 2014 [73]	A, B, D	E, F, H	I	7
Hartvigsen el al, 2003 [68]	A, B, C, D			4
Kristofferson et al, 2012 [20]	A, B,	E, F, G	I	6
Kristoffersen et al, 2013 [79]	A, B, D	E, F,	I	6
Lambert et al, 2010 [77]	A, D	F, G, H	I	6
Lyngberg et al, 2005 [1, 52]	A, B, C, D	E, F		6
Malone et al, 2015 [71]	B, C, D	F,		4
Ossendorf et al, 2009 [60]	A, B, C, D	F, H	I	7
Rossi et al, 2005 [53]	A, B, D	E, F, H,	I	7
Rossi et al, 2006 [58]	A, B, D, E, F, H		I	7
Rossi et al, 2008 [59]	A, B, C, D	E, F, H		7
Rubinstein et al, 2000 [67]	A, B, C, D	F, H		6
Sanderson et al, 2013 [21]	A, B, C, D	E, F, G, H		8
S von Peter et al, 2002 [78]	C, D	E, F, G, H	I	7
Vukovic et al, 2010 [48]	A, B, C, D	E, F,		6
Wells et al, 2010 [51]	A, B, D	F, G, H		6
Wells et al, 2011 [50]	A, B, D	F, G, H	I	7
Xue et al, 2008 [55]	A, B, D	F, G, H		6

Key: A-Sampling reported, B-Sample size >100, C-Response rate >75%, D-Low recall bias, E-Classification of headache type, F-Age and sex, G-Ethnicity, H-Socioeconomic status Scoring: 1-4 poor quality, 5-6 low quality, 7-8 moderate quality, 9-10 high quality

## Results

The key findings of the 35 articles were grouped and evaluated using a critical review approach adapted from previous research [46, 47]. Based on the limited information available for other headache types, prevalence findings are reported within one of two categories - either as ‘migraine’ for papers reporting studies where the population was predominately or entirely made up of migraine patients or as ‘headache’ for papers where the study population was predominately other headache types (including tension-type headaches, cluster headaches, cervicogenic headache) and/or where the headache type was not clearly stated. Ten papers reported findings examining prevalence rates for the ‘migraine’ category alone, 18 papers reported findings examining prevalence for the ‘headache’ category alone and 3 papers reported findings for both categories. Based on the nature of the information available, prevalence use was categorised by manual therapy providers. The extracted data was then analysed and synthesized into four thematic categories: *prevalence; profile and motivations for MT use; concurrent use and order of use of headache providers; and self-reported evaluation of MT treatment outcomes.*

### Prevalence of MT use

Thirty-one of the reviewed articles with a minimum sample size (>100) reported findings regarding prevalence of MT use. The prevalence of chiropractic use for those with migraine ranged from 1.0 to 36.2% (mean: 14.4%) within the general population [19–21, 48–52] and from 8.9 to 27.1% (mean: 18.0%) within headache-clinic patient populations [53, 54]. The prevalence of chiropractic use for those reported as headache ranged from 4 to 28.0% (mean: 12.9%) within the general population [20, 48, 51, 55–57]; ranged from 12.0 to 22.0% (mean: 18.6%) within headache/pain clinic patient populations [58–60] and from 1.9 to 45.5% (mean: 9.8%) within chiropractic patient populations [61–69].

The prevalence use of physiotherapy for those with migraine ranged from 9.0 to 57.0% (mean: 24.7%) within the general population [19, 20, 48, 52] and from 4.9 to 18.7% (mean: 11.8%) within headache-clinic patient populations [54, 70]. The prevalence use of physiotherapy for those reported as headache ranged from 12.2 to 52.0% (mean: 32.1%) within the general population [20, 48] and from 27.8 to 35.0%% (mean: 31.4%) within headache/pain clinic populations [60, 70].

Massage therapy use for those with migraine ranged from 2.0 to 29.7% (mean: 15.6%) within the general population [49, 50, 71] and from 10.1 to 56.4% (mean: 33.9%) within headache-clinic populations [53, 54, 72, 73]. Massage/acupressure use for those reported as headache within headache/pain clinic patient populations ranged from 12.0 to 54.0% (mean: 32.5%) [58–60, 70].

Osteopathy use for those with migraine was reported as 1% within the general population [49]; as 2.7% within a headache-clinic patient population [53] and as 1.7% within an osteopathy patient population [74]. For headache the prevalence was 9% within a headache/pain clinic population [60] and ranged from 2.7 to 10.0% (mean: 6.4%) within osteopathy patient populations [74, 75].

The combined prevalence rate of MT use across all MT professions for those with migraine ranged from 1.0 to 57.0% (mean: 15.9%) within the general population; ranged from 2.7 to 56.4% (mean: 18.4%) within headache-clinic patient populations and was reported as 1.7% in one MT patient population. The combined prevalence rate of MT use across all MT professions for those reported as headache ranged from 4.0 to 52.0% (mean: 17.7%) within the general population; ranged from 9.0 to 54.0% (mean: 32.3%) within headache-clinic patient populations and from 1.9 to 45.5% (mean: 9.25%) within MT patient populations.

### Profile and motivations for MT use

While patient socio-demographic profiles were not reported within headache populations that were exclusively using MT, several studies report these findings where MT users made up a significant percentage of the non-medical headache treatments utilized by the study population (range 40% – 86%: mean 63%). While findings varied for level of income [58, 70] and level of education, [70, 72, 73] this patient group were more likely to be older [70, 72], female [20], have a higher rate of comorbid conditions [58, 70, 76] and a higher rate of previous medical visits [20, 58, 70] when compared to the non-user group. Overall, this group were reported to have a higher level of headache chronicity or headache disability than non-users [20, 54, 58, 70, 72, 77].

Several studies within headache-clinic populations report patient motivations for the use of complementary and alternative headache treatments where MT users made up a significant proportion of the study population (range 40% – 86%: mean 63%) [58, 70, 72, 78]. From these studies the most common motivation reported by study patients was ‘seeking pain relief’ for headache which accounted for 45.4% – 84.0% (mean: 60.5%) of responses. The second most common motivation was patient concerns regarding the ‘safety or side effects’ of medical headache treatment, accounting for 27.2% – 53.0% (mean: 43.8%) of responses [58, 70, 72]. ‘Dissatisfaction with medical care’ accounted for 9.2% – 35.0% (mean: 26.1%) of responses [58, 70, 72].

A limited number of reviewed papers (all from Italy) report on the source of either the referral or recommendation to MT for headache treatment [53, 58, 59]. From these studies, referral from a GP to a chiropractor ranged from 50.0 to 60.8% (mean: 55.7%), while referral from friends/relatives ranged from 33.0 to 43.8% (mean: 38.7%) and self-recommendation ranged from 0 to 16.7% (mean: 5.6%). For massage therapy, referral from a GP ranged from 23.2 to 50.0% (mean: 36.6%), while referral from friends/relatives ranged from 38.4 to 42.3% (mean: 40.4%) and self-recommendation ranged from 7.7 to 38.4% (mean: 23.1%). For acupressure, referral from a GP ranged from 33.0 to 50.0% (mean: 41.5%), while referral from friends/relatives was reported as 50% and self-recommendation ranged from 0 to 16.6% (mean: 8.3%). One study reported findings for osteopathy where referral from both GP’s and friends/relatives was reported as 42.8% and self-recommendation was reported as 14.4%. Overall, the highest proportion of referrals within these studies was from GPs to chiropractors for chronic tension-type headache (56.2%), cluster headache (50%) and migraine (60.8%).

### Concurrent use and order of use of headache providers and related communication of MT users

Several studies report on the concurrent use of medical headache management with complementary and alternative therapies. In those studies where the largest percentage of the patient population were users of MT’s (range 57.0% – 86.4%: mean 62.8%), [58, 70, 78] concurrent use of medical care ranged between 29.5% and 79.0% (mean: 60.0%) of the headache patient population.

These studies further report on the level of patient non-disclosure to medical providers regarding the use of MT for headache. Non-disclosure ranged between 25.5 and 72.0% (mean: 52.6%) of the patient population, with the most common reason for non-disclosure reported as the doctor *‘never asking’,* ranging from 37.0 to 80.0% (mean: 58.5%). This was followed by a patient belief that *‘it was not important for the doctor to know’* or *‘none of the doctor’s business’,* ranging from 10.0 to 49.8% (mean: 30.0%). This was followed by a belief that either *‘the doctor would not understand’* or *‘would discourage’* these treatments, ranging from 10.0 to 13.0% (mean: 11.5%) [53, 77].

One large international study reported the ordering of the typical provider of headache care by comparing findings between several countries for migraine patients [21]. Primary care providers followed by neurologists were reported as the first and second providers for migraine treatment for nearly all countries examined. The only exception was Australia, where those with chronic migraine selected chiropractors as typical providers at equal frequency to neurologists (14% for both) while those with episodic migraine selected chiropractors at a greater frequency to neurologists (13% versus 5%). Comparatively, chiropractors were selected as the typical provider for those with chronic migraine by 10% in USA and Canada, 1% in Germany and 0% for UK and France. Chiropractors were selected as the typical provider for those with episodic migraine by 7% in USA, 6% in Germany, 4% in Canada and by 1% in both the UK and France.

#### Self-reported effectiveness of MT treatment outcomes

Several headache and pain-clinic population studies provide findings for the self-reported effectiveness of MT headache treatment. For chiropractic, patient self-reporting of partially effective or fully effective headache relief ranged from 27.0 to 82.0% (mean: 45.0%) [53, 58–60, 78]. For massage therapy, patient self-reporting of partially effective or fully effective headache relief ranged from 33.0 to 64.5% (mean: 45.2%)[53, 58, 60, 73, 78], and for acupressure this ranged from 33.4 to 50.0% (mean: 44.5%) [53, 58, 59]. For osteopathy and physiotherapy, one study reported effectiveness as 17 and 36% respectively [60].

When results are combined across all MT professions the reporting of MT as either partially or fully effective ranged from 17.0 to 82.0% (mean 42.5%) [53, 58–60, 73, 78]. In addition, one general population study provides findings for the self-reported effectiveness for chiropractic and physiotherapy at 25.6 and 25.1% respectively for those with primary chronic headache and 38 and 38% respectively for those with secondary chronic headache [79].

## Discussion

This paper provides the first critical integrative review on the prevalence and key factors associated with the use of MT treatment for headaches within the peer-reviewed literature. While study methodological limitations and lack of data prevent making strong conclusions, these findings raise awareness of issues of importance to policy-makers, educators, headache providers and future research.

Our review found that MT use was generally higher within medical headache-clinic populations when compared to general populations. However, the use of individual MT providers does vary between different regions and this is likely due to a number of factors including variation in public access, healthcare funding and availability of MT providers. For example, the use of physiotherapy for some headache types may be relatively higher in parts of Europe [20, 60] while the use of chiropractors for some headache types may be relatively higher in Australia and the USA [19, 21]. Overall, the prevalence use of MT for headache appears to be substantial and likely to be the most common type of physical therapy utilized for headache in many countries [19–21, 49]. More high quality epidemiological studies are needed to measure the prevalence of MT use across different headache types and sub-types, both within the general population and clinical populations.

Beyond prevalence, data is more limited regarding who, how and why headache patients seek MT. From the information available however, the healthcare needs of MT headache patients may be more complex and multi-disciplinary in nature compared to those under usual medical care alone. Socio-demographic findings suggest that users of MT and other complementary and alternative therapies have a higher level of headache disability and chronicity compared to non-users. This finding may correlate with the higher prevalence of MT users within headache-clinic populations and a history of more medical appointments. This may also have implications for future MT trial designs both in terms of the selection of trial subjects from inside versus outside MT clinical settings and the decision to test singular MT interventions versus MT in combination with other interventions.

Limited information suggests that a pluralistic approach toward the use of medical and non-medical headache treatments such as MT is common. While findings suggest MT is sought most often for reasons of seeking headache relief, the evidence to support the efficacy of MT for headache relief is still limited. MT providers must remain mindful of the quality of the evidence for a given intervention for a given headache disorder and to inform patients where more effective or safer treatment interventions are available. More research is needed to assess these therapies individually and through multimodal approaches and for studies to include long-term follow-up.

Information limited to Italy, suggests referral from GPs for MT headache treatment can be common in some regions, while this is less likely to widespread given the issue of patient non-disclosure to medical doctors regarding the use of this treatment in other studies. High quality healthcare requires open and transparent communication between patients and providers and between the providers themselves. Non-disclosure may adversely influence medical management should unresponsive patients require further diagnostic investigations [80] or the implementation of more effective approaches to headache management [81] or prevents discussion in circumstances where MT may be contraindicated [82]. Primary headache providers may benefit from paying particular attention to the possibility of non-disclosure of non-medical headache treatments. Open discussion between providers and patients about the use of MT for headache and the associated outcomes may improve overall patient care.

### Future research

Despite the strong need for more high quality research to assess the efficacy of MT as a treatment for headache, the substantial use of MT brings attention to the need for more public health and health services research within this area of headache management. The need for this type of research was identified in a recent global report on the use of headache-related healthcare resources [18]. Furthering this information can lead to improvements in healthcare policy and the delivery of healthcare services.

The substantial use of physical therapies such as MT has been under-reported within many of the national surveys reporting headache-related healthcare utilization [3, 5, 83–85]. Regardless, the role of physical therapies in headache management continues to be assessed, often within mainstream and integrated headache management settings [86–89]. Continuing this research may further our understanding of the efficacy and outcomes associated with a more multidisciplinary approach to headache management.

Further to this is the need for more research to understand the healthcare utilization pathways associated with those patients who use MT in their headache management. Little is known about the sociodemographic background, types of headaches, level of headache disability and comorbidities more common to this patient population. In turn, such information can provide insights that may be valuable to provider clinical decision-making and provider education.

### Limitations

The design and findings of our review has a number of limitations. The design of the review was limited by a search within English language journals only. As a result, some research on this topic may have been missed. While the quality scoring system adopted for this review requires further validation, the data we collected was limited by the low to moderate quality of available papers which averaged 6.4 out of 10 points (Table 3). The low scoring was largely due to significant methodological issues and the small sample size associated with much of the collected papers. Much of the data on this topic was heterogeneous in nature (telephone, postal surveys and face-to-face interviews). There was a lack of validated practitioner and patient questionnaires to report findings, such as for questions on prevalence, where the time frames utilized varied between ‘currently’, ‘last 12 months’ and ‘ever’.

Data on the prevalence of MT use for headache was limited particularly within individual MT provider populations when compared to data found within the general population and headache-clinic populations. Many studies assessed the use of MT for headache without identifying headache types. Only one study inside an MT population had reported the percentage of patients attending for reasons of migraine alone (osteopathy). The prevalence of MT use for headache was reported most within chiropractic patient population studies, however information was limited on the types of headache. We found no studies reporting the prevalence of headache patients within physiotherapy or massage therapy patient populations using our search terms.

A lack of data for some themes necessitated providing findings pooled with users of other non-medical headache providers. Data within many geographical regions was very limited with the most limited data was on the source of referral to MT headache providers (three papers from Italy only). These limitations support the call for more research to be focused exclusively within MT populations and different regional areas before stronger conclusions can be drawn.

## Conclusion

The needs of those with headache disorders can be complex and multi-disciplinary in nature. Beyond clinical research, more high quality public health and health services research is needed to measure and examine a number of issues of significance to the delivery and use of MT’s within headache management. With unmet needs still remaining for many who suffer recurrent headaches, clinicians should remain cognizant of the use of MT’s and remain open to discussing this approach to headache management in order to ensure greater safety, effectiveness and coordination of headache care.

## Abbreviations

MT: Manual therapy
EMG: Electromyography
